# Barriers to Accessing Health Care in Rural Regions by Transgender, Non-Binary, and Gender Diverse People: A Case-Based Scoping Review

**DOI:** 10.3389/fendo.2021.717821

**Published:** 2021-11-18

**Authors:** Janis Renner, Wiebke Blaszcyk, Lars Täuber, Arne Dekker, Peer Briken, Timo O. Nieder

**Affiliations:** Institute for Sex Research, Sexual Medicine and Forensic Psychiatry, University Medical Center Hamburg-Eppendorf, Hamburg, Germany

**Keywords:** trans health care, barriers accessing health services, transgender mental health, geographic location, urban-rural divide, remoteness, e-health

## Abstract

Research shows an overrepresentation of trans people in vulnerable socioeconomic situations, primarily due to experiences of discrimination. At the same time, rural or suburban living areas often lack specialized trans-related health care, which a majority of trans people rely on to some extent. Taken together, the lack of both socioeconomic resources and access to trans-related health care can exacerbate health-related distress and impairment for trans people. We illustrate this problem using case vignettes of trans people from rural and suburban areas in (Northern) Germany. They are currently participating in an e-health intervention and randomized controlled trial (RCT) called *i^2^TransHealth*, whose case vignettes provided the impetus for the scoping review. The scoping review analyzes the impact of place of residence and its intersection with barriers to accessing trans-related health care. PubMed and Web of Science Data bases were searched for relevant studies using a search strategy related to trans people and remote, rural, or suburban residences. 33 studies were selected after full-text screening and supplemented *via* reference list checks and study team expertise by 12 articles addressing the living conditions of remotely living trans people and describing requirements for trans-related health care. The literature on trans people living remotely reveals intersections of trans mental health with age, race, gender expression, geographic location, community size, socioeconomic status, discrimination experiences, and attitudes towards health care providers. Several structural health care barriers are identified. The role of health care professionals (HCPs) for remotely living trans people is discussed. There is no need assuming that rural life for trans people is inevitably worse for health and well-being than urban life. Nevertheless, some clear barriers and health disparities exist for trans people in remote settings. Empowering trans groups and diversity-sensitive education of remote communities in private and institutional settings are needed for respectful inclusion of trans people. Facilitating access to trans-related health care, such as through video-based e-health programs with HCPs, can improve both the health and socioeconomic situation of trans people.

## Introduction

Living outside of metropolitan areas often presents a significant challenge when trying to access specialized health care. In particular, health care for trans people (the term “trans” includes but is not limited to transgender, non-binary, or gender diverse people) remains a service provided mainly in larger cities. Thus, finding health care that meets their needs can often prove difficult for trans people living in rural settings since trans-informed health care professionals (HCPs; e.g., mental health professionals, in short MHPs, or physicians) are unlikely to be present in the area (1). Accessing health care becomes more complicated when certain factors, such as age, financial insecurity, or lack of education, are added to the difficulties inherent in rural areas. Improving this situation could be beneficial for trans people, as research has shown that good experiences with HCPs are positively associated with both general and mental health (2).

E-health approaches are being considered as a possible solution for access barriers to trans-related health care (3, 4). They can provide appropriate mental and physical health services to a wider range of people (5, 6). As a broad application area, e-health means a range of technologies to promote health and well-being. It ranges from electronic patient records and online consultations to mobile devices or apps. In short, e-health functions as a collective term for electronic information and communication systems in the health care system (7, 8). HCPs offer services using digital software with the aim of supplementing and improving their services. E-health approaches specific to mental health are often discussed interchangeably with the terms online therapy or distance counseling, implying treatment despite physical distance. Here, HCPs use electronic media enabling digital exchange with their patients, whether through e-mail, chat or video consultations (4). While digital care was long understood to mean primarily electronic patient records (7), newer services such as video consultations are gradually gaining ground in the course of digitalization and the legalization of communication media for patient treatment. E-health platforms that include video consultations still require intensive evaluation because they are a relatively novel tool (8). However, e-health approaches could be particularly helpful for hard-to-reach groups. Specifically, e-health approaches including video consultations are meant to help trans people accessing trans-informed HCPs regardless of their place of residence. In order to further investigate the potential of e-health approaches, we are currently conducting a randomized controlled trial (RCT) that allows trans people in remote, rural, or suburban areas in the early phase of transition or exploration of gender-related issues to participate in our internet-based health care program *i²TransHealth* (https://www.i2transhealth.de/english-landing-page/), which provides video consultations by trans-informed HCPs and local crisis interventions by general practitioners (GPs) and psychiatrists.

In the course of clinical work with service users of our internet-based health care program, *i²TransHealth*, it has become clear that trans people away from metropolitan areas experience disadvantages due to where they live. They experience marginalization through close-knit, prejudiced rural or small-town communities, they distrust their surroundings, and some see trans-related health care in their area as critical or nonexistent. We sought to examine this clinical impression and identify evidence-based studies that allow meaningful judgments about the additional burden of rural socialization of trans people on access to trans-related health care. A 2016 systematic review generally addressed primary health care for trans people with almost exclusively urban samples (9). A 2017 systematic review summarized rural sexual and gender minority health and health care generally without a trans-specific focus (10). Both systematic reviews examined U.S. studies exclusively (9, 10). Thus, we analyzed specific barriers to accessing trans-related health care of trans people with rural residential experiences. We collected clinical impressions in case vignettes and used this as a basis to develop a case-based scoping review that specifically addresses rural socialization of trans people, including those outside the United States.

This article reviews the existing literature on the lives of trans people in relation to their place of residence (e.g., remote, rural, and suburban areas) and how this, in addition to other sociodemographic factors and multiple lived experiences of discrimination in the community at home and among HCPs, affects access to trans-related health care. If studies permit a statement, differences and similarities between rural areas and big cities are outlined. First, we introduce case vignettes that aim at illustrating the situation of three trans people living in rural or suburban areas and are currently participating in *i^2^TransHealth*. The case vignettes appear different at first glance, but their shared problems stem from their remote living situation and have thus inspired this paper. As the case vignettes reveal intersecting barriers which require closer examination, they served as a guiding lens through which we reviewed the recent research on the topic.

The first case vignette of a trans woman illustrates that disclosure of her female gender in late adulthood life can be difficult because small-town, close-knit social structures can be rigidly attached to one form of living together:

*M., a 60-year-old Caucasian trans woman with a middle school education, does the domestic work in her small family and lives in a small town. Encouraged by the emerging social liberalization, she no longer wanted to hide and, after consulting her wife, occasionally put on her “feminine” clothes when alone with her. She now increasingly expresses her femininity in everyday life at home, but since she doesn’t want other people in town to see her this way, she changes clothes and removes make-up several times a day. The increased female gender expression has led to resistance from M.’s wife, who has said “I married a man” and has threatened to move out. She currently has no contact with other trans people nor is she trying to engage with the trans community. M. understands how overwhelming her transition might be for her family. In order to make them less uncomfortable, she wears less explicitly feminine clothes or does so only during the video consultations conversations. At one point, she dared to go to the nearby city dressed in feminine clothes but disguised by a face mask (due to COVID-19). She wishes she could go outside like this with her wife but can currently only express her female gender at home and even there it is limited. Thus, the current goal of treatment remains searching for ways, places and the right pace at which M. can live as the woman she is, while at the same time not threatening her family’s cohesion.*

Another aspect of a remote life may be a lack of knowledge about gender dysphoria, and thus an awareness of treatment options and potential pathways must first be acquired:

*L. is a 26-year-old Caucasian trans man living in a village in Northern Germany. As a child, L. always insisted on short hairstyles and displayed several traditionally male-associated interests and hobbies. For a long time, L. did not understand why he felt such intense discomfort in respect to his body. Only three years ago did he stumble onto a YouTube video by a fellow trans man and learned about the concept of being transgender. Few MHPs work within L.’s local area and even fewer have sufficient knowledge about gender dysphoria. Prior to joining the i^2^TransHealth project, L. had one initial session with a MHP but didn’t feel comfortable there. The internet has been L.’s main source of information about trans issues, but he doesn’t like posting about his personal matters in online forums or on social media. Given the lack of local in-person opportunities, his options of meeting and talking to people who share some of his experiences remain very limited.*

The third case vignette shows that the consequences of discriminatory experiences in health care can lead to significant impairment of one’s health:

*R., a 21-year-old Caucasian trans man, lives on a farm in a small town. From early childhood he has felt “more like a boy than a girl”. Several years ago, R. sought out a MHP to move forward with his medical transition. However, he felt extremely uncomfortable with this MHP who frequently invalidated his experience. R. felt patronized and belittled. He says the MHP simply didn’t believe him when he told him about his gender experience. Consequently, he also did not receive hormonal treatment. After a year, R. quit therapy. He says that this MHP was more harmful than helpful and that this experience put him off seeking further medical or mental health care for a long time. It took R. two years to recover from this. Only then did he find the courage to try again by reaching out to the i^2^TransHealth project. Having to live in a body widely perceived as female severely limits R.’s self-expression. He is constantly self-monitoring his appearance and his effect on other people and feels forced to “perform” his masculinity much more stereotypically than a cis man would have to. R. does not want to start his professional training by having to out himself in front of so many people (again) and is thus waiting until hormone treatment has begun and shown some effects.*

As illustrated by the case vignettes, barriers to care are a frequently discussed problem in trans health services (5, 11). However, the actual characteristics of, and differences between, trans-related health care in urban and remote settings have rarely been researched (12). With this in mind, we aim to identify and reduce research gaps concerning the remote, rural, or suburban situation of trans people. Therefore, we review and evaluate previous research on the impact of rural or suburban living on access to general and trans-related health care, and identify research questions to be investigated in future studies. Additionally, we discuss how to better address trans people’s needs in rural areas by taking an intersectional view of their experiences within the health care system. Thus, we aim to answer the following research questions:

1. How does living in rural areas affect access to specialized trans-related health care services?
2. How do health burdens of trans people in rural areas intersect with other barriers and risk factors in health care?
3. What possible solutions have been identified to address the problem of health burdens of trans people in rural areas?

## Materials and Methods

Scoping reviews have proven useful for research questions for which large research gaps and sparse literature available for systematic analysis (13, 14). The methodology of a scoping review is appropriate for providing guidance on the current state of the research literature in the case of a paucity of research on a topic and for making recommendations for future research (14). A scoping review aims to delineate previous concepts and substantive boundaries on an area of research by including studies regardless of their quality and allowing for an up-to-date assessment of the evidence (14).

For the present scoping review, the objective was to examine the significance of the urban-rural divide for trans-related health care. The overarching lens culminates in the three research questions of whether rural environments impose health burdens on trans people, whether these potential health burdens overlap with barriers to health care access that have already been studied in more detail, and whether health burdens can be prevented or reduced in non-urban communities. As introduced by the case vignettes, the three research questions of the scoping review address the impact of trans people’s remote, rural, or suburban socialization experience on their barriers to accessing trans-related health care and the interrelated aggravating factors. Against the background of the case vignettes, we analyze the evidence on residence as an aggravating factor combined with other barriers to care. This case-based scoping review concentrates on research investigating the benefits and drawbacks of rural living for trans people and/or their specific barriers to care. More general research on the lives of trans people is also discussed.

Within the present scoping review, the target population are trans people regardless of age or identity, although combined LGBTQ+ samples with trans people (i.e., people who identify as lesbian, gay, bisexual, transgender, or queer; the + stands for the inclusive representation of all identities and expressions) were allowed in the absence of comprehensive research on the topic area. Additionally, HCPs were included in the target population if they provided information about their work with trans people. Articles were excluded if they generally lacked any meaningful findings on non-urban residences or specifically failed to provide more in-depth analyses to trans people in combined samples. The core concept examined was remote, rural, or suburban regions and their intersection with barriers to accessing trans-related health care. The specific context was explicitly geographic location and thus the extent to which remote, rural, or suburban socialization influences trans health. Studies should consider perspectives of trans people with past or current rural residential experiences.

The search strategy followed a 3-step process recommended for a scoping review (14). First, a non-systematic search of databases was conducted to determine if studies existed on the topic under investigation. Based on the key terms found in abstract or full text, we found that an overly differentiated, potentially limiting search strategy was not indicated given the paucity of research. Second, a search was performed in the PubMed and Web of Science databases using the terms “transgender” AND “rural”. During the review process, the search string was adapted to include synonyms related to transgender and rural areas. As we assumed the evidence base to be weak, no further restrictions were made. Third, the reference lists of the selected papers in the full-text review were scanned for additional potentially relevant studies. The approach to the search strategy was iterative in design, thus ensured an overview of the literature, a critical review of the search strategy, and supplementation of the included study through repeated searches. The full search strategy can be found in **Appendix 3** (**Supplementary Material**).

Both first and last author (JR, TN) elaborated on the search strategy and made considerations about specificity and sensitivity of the search. Due to the sparse literature and in order not to exclude relevant studies, we applied a high sensitivity to the search strategy, i.e., including false positive hits irrelevant to the research question was preferred over excluding relevant papers. JR exported the citations, pre-selected relevant studies in Rayyan (15), and performed full-text screening. The search included empirical qualitative and quantitative articles as well as theoretical reviews on the general situation and mental health of trans people in rural areas. Based on the research group’s expertise on barriers to accessing trans-related health care, six other empirical studies and reports relevant to the research question were included. JR and TN discussed the reasonable inclusion of further studies for this purpose. In the course of this, included information sources went beyond empirical studies. Thus, reviews, reports, and gray literature are also found in the present scoping review (14).

The combined database searches from PubMed and Web of Science yielded 497 records (see **Appendix 1 Figure 1, Supplementary Material**). Of these, 133 duplicate records were removed before review. JR reviewed the papers according to the following inclusion criteria: peer-reviewed publication in English by mid-August 2021, study participants included HCPs or trans people, possibly also as a subgroup of the LGBTQ+ community, and non-urban place of residences were explicitly addressed. Research group members read articles in full if these criteria were met. 77 citations were included in the full-text review and checked for eligibility. The present work complies with the extension of the PRISMA Statement for Scoping Reviews (PRISMA-ScR) (16).

## Results

For the scoping review, 33 records were identified, to which additional literature was added. Supplemented by studies taken from reference lists and papers known to the research group, a total of 45 sources (43 empirical studies, 2 reviews) were selected for the scoping review and are presented in **Table 1** (**Appendix 2, Supplementary Material**). Among the 43 empirical studies, we found two types of articles: 31 articles dealing explicitly with trans people and 12 articles dealing with trans people within the general or broader LGBTQ+ population. The included studies are divided by study type into 28 quantitative studies (including 23 original papers, 4 reports, 1 poster presentation), 13 qualitative studies, 2 mixed method studies, and 2 systematic reviews. A flowchart of the study selection process is shown in **Figure 1** (see **Supplementary Material**). The final 45 articles included in the review describe studies from the United States, Canada, Australia, Germany, Georgia, Poland, Serbia, Spain, and Sweden, as well as an overall report on the situation in the European Union including the United Kingdom.

The few studies of trans people who have had experiences living in rural areas generally show mixed results such as influences from rural socialization experienced to varying degrees by trans people. To further organize the findings, we also looked at studies that consider trans people in the larger context of access barriers to trans-related health care, where the variable of place of residence is described but not always one of the main factors discussed.

Given the three research questions of the scoping review, we illustrate the results to clarify the influence of the urban-rural divide on access to trans-related health care. We begin with the sociodemographics of a trans person that regulate their access to health care (research question 1). We follow this with intersecting factors such as discrimination in the community and highlight the consequences of negative or inadequate care situations as a health risk (research question 1 and 2). Previous approaches to addressing existing problems, such as support persons or groups and training for HCPs and caregivers, are listed in the following sections (research question 3).

### Sociodemographic Aspects Concerning Trans People

In order to answer the first research question, sociodemographic data can provide an initial overview of possible health burdens for trans people due to their place of residence. The literature search detected several sociodemographic aspects that shape the lives of trans individuals and their access to trans-related health care. In terms of socioeconomic status, trans people are as likely to be married, employed and living in a rural area as the rest of the population, but are more likely to be People of Color, below the poverty line, and without a college degree according to a household probability sample of U.S. adults with 691 trans adults compared to 150,765 cis adults across all age groups (17). Several U.S. surveys confirm that many trans people, as well as other members of LGBTQ+ communities, do not have health insurance given their financial challenges and lack of mandatory coverage (18, 19). In terms of housing, a substantial number experience homelessness; in the U.S. National Transgender Survey of 27,715 respondents of all ages 18 and older, 30% ever experienced homelessness and 12% experienced homelessness in the past year (19). The LGBTI II report of the European Union member states and United Kingdom with over 139,799 people (mean age 29, age range 15 to 55+; 14% of the sample are trans people) shows 7% of European trans people are unemployed, 5% are unable to work due to health reasons, 46% have some to great difficulty making ends meet in their households, and a total of 48% report their place of residence as small town, village, or home in the countryside (11).

Problems stemming from sociodemographics come to a head for People of Color in limited access to health care in the U.S. According to a large quantitative study of 5,135 US transgender veterans (mean age 51.21), Black compared to White transgender veterans delayed or did not use mental health services even when they existed (20). At the same time, Black transgender veterans are 65% less likely to live in rural areas (20). But they have greater social disadvantages, increased health risk, be it alcohol abuse, heart problems, high blood pressure or depression (20).

Age is also a critical variable for trans patients seeking treatment. Given an online survey of 252 respondents (mean age 47.9), rural residing trans and LGBTQ+ US veterans face longer travel times to HCPs than their suburban or urban counterparts (21). According to the Canada Trans Health Survey, for rural and remote residing trans youth in particular, who comprised 9.3% of the total sample, aged 14 to 25, transportation presents significant obstacles for accessing urban health care (2). Samples often focus on middle-aged participants, neglecting trans youth in rural or suburban areas, where sparse research exists (22). However, trans people at the lower and higher ends of the age spectrum are particularly isolated in regards to health care (2, 21).

### Growing up and Living With Bullying and Discrimination in the Community

The first two research questions require data on health burdens of rural residential experiences and how these, along with other barriers to trans-related health care, contribute to the extent to which help is sought and trusted by HCPs. The included studies reveal that the social climate of a rural or suburban community in which a person grows up or lives is formative in one’s life, making sexual and gender minorities (SGM) vulnerable to mental health issues and less likely to seek help in their community or health care. A systematic review highlights that isolation in rural areas and low levels of social support have a negative impact on the health of SGM, and discusses the sparse health data and lack of tailored interventions to address existing disadvantages for LGBTQ+ people in remote, rural areas (10).

A school-based survey in the US state of Minnesota with 2,168 trans youth in 9^th^ and 11^th^ grade, representing middle adolescent age groups, found that those living in rural areas reported the highest levels of bullying and victimization compared to urban areas, while emotional distress was highest among people living in suburban areas (22). For LGBTQ+ people, an online survey in the US state of Nebraska with 770 respondents (aged 19 to 60 years or older; 10.9% of respondents identified as transgender) showed that rural LGBTQ+ people engaged less with others socially, came out to fewer people, and showed lower self-acceptance compared to their urban counterparts (18). A U.S.-wide survey of 5,420 LGBTQ+ secondary students (mean age 15.9 years; of whom 4.5% identified as transgender and 4.0% with a different gender identity) revealed that, as a subgroup, transgender youth are more likely to be bullied for their gender expression or sexual orientation than gay or bisexual male youth (23). Although students in rural schools generally experience less violence and harassment than general population in urban schools, LGBTQ+ youth experience rural schools as extremely unsafe places due to not blending in with cis-heteronormative norms (23). Conversely, queer youth in urban regions are less likely to experience discrimination than their rural counterparts. Victimization at school is often associated with increased self-harming or risky health behaviors among LGBTQ+ youth (23), compounded by a lack of safe spaces in a more homogenous rural school community or an environment that is generally less diverse. According to qualitative interviews with 30 trans adults (mean age 36.0) in rural US state of Montana, rural residing trans people have to deal with bullying, discrimination and marginalization in their environment (24). In particular, suicidality rates are high there. 80.0% reported having had suicidal thoughts in the past, and 46.7% reported having attempted suicide in the past (24). According to qualitative in-depth interviews with 19 trans people between the ages of 15 and 22 (mean age 18) living in Midwestern U.S. states, they indicate that resources for trans people such as existing SGM community groups and previous support or external validation of sexual or gender identity make them feel comfortable in the social climate of their area (25). According to data from 14 to 18 year old trans and gender questioning youth from 7 qualitative in-person interviews and a survey of 70 respondents in the Midwestern U.S., young trans people’s estimations of rural community climates toward SGM range from unsupportive to hostile (26). In rural areas, such as the US state of Nebraska, trans people tend to be less involved in peer groups and less supported by family or friends compared to other groups within the LGBTQ+ spectrum (18). Primary caregiver support is critical to the experience of social support because it can foster appreciative, respectful interactions within the family and environment, as revealed by in-depth interviews about maternal support with 25 trans adults (mean age 34.48) in Central Appalachia (27). MHPs, the authors recommend, should include the closest caregivers *via* counseling or psychoeducational workshops in rural areas to address the potential for stigma and stress within small and insular social networks (27). According to qualitative interviews with US trans people (age range 25 to 61; a focus group of 6 persons and 1 individual interview), interrelated issues affecting their well-being that MHPs should address are vocation, personal change and coming-out, acceptance, and identity (28). In these aspects, trans individuals are highly dependent on the support of their family and environment, which has often experienced less education on gender fluidity than urban spaces (28). In a qualitative interview study of 25 trans women (mean age 27.56 years) from the U.S. state of Oregon, several trans women who moved to Oregon’s metropolitan areas with rural residential experience in Oregon or neighboring states addressed prior victimization experiences in general or severe threats of violence specifically in the family (29). Later, the “family of choice” in adulthood is seen as playing an important role in empowerment, navigating health care systems, and arrival in a metropolitan trans-friendly community (29).

According to an intersectional analysis of 45 individual interviews with trans men from the U.S. Midwest and Southeast, trans people do not reject rural life per se, but many do not want to stay in their home region but move to a new rural area in order to avoid unpleasant encounters with former acquaintances (30). A qualitative study from a Canadian small city with 13 young LGBTQ+ participants (aged 15 to 25 years; 4 individual interviews and 2 focus groups), of which 5 were trans, indicated that LGBTQ+ people who had so far lived exclusively in rural areas considered small towns to be more restrictive than LGBTQ+ people who had experiences living in both rural and urban areas (12).

### Rural Health Care Providers

Previous research on stakeholders’ estimations of LGBTQ+ populations by 207 community members from various U.S. town hall dialogues and summits revealed knowledge deficits in HCPs and a lack of culturally sensitive expertise in rural areas (31). Past research examining treatment counselors’ attitudes toward trans people and LGB people found equal deficits in knowledge or skills to provide competent help in rural and urban settings (32). In 2004, 109 counselors from urban Chicago (mean age 41.3) and 242 counselors from rural Iowa (mean age 40.9), were similar in terms of their increased negative attitudes toward trans people (32).

The unquestioned assumption that patients are cisgender and heterosexual create a discriminatory experience for many LGBTQ+ people (33). From various survey and interview data with HCPs and LGBTQ+ people from the United States, Canada, and Europe (33–35), it appears that various HCPs exhibit ambivalence toward LGBTQ+ patients, microaggressions, and microinvalidations, e.g., misgendering or deadnaming trans clients. Rural HCPs have fewer LGBTQ+ patients and less diversity-related training opportunities, making the conscious creation of an LGBTQ+ friendly environment more unlikely (31). This, combined with the close-knit nature of many rural communities, makes it difficult to be open about one’s gender identity or sexual orientation to a rural HCP because they probably know one’s relatives and friends, according to qualitative in-depth interviews with 16 LGBTQ+ youth (ages 15-24) and 21 LGBTQ+ adults (ages 25 or older), as well as 14 key informants with experience working with LGBTQ+ clients in the Northwest Territories, Canada (33).

Both the Trans Health Survey in Europe (surveyed 885 trans health care users ages 16-77 with a mean age of 27 and 888 HCPs with mean age 41.7 from Georgia, Poland, Serbia, Spain, and Sweden) and national data from 5,831 U.S. transgender adults (mean age 37.0) show that when the environment is perceived as discriminatory rather than inclusive, the odds of trans people believing in and seeking trans-related health care are low (35, 36). A German online survey with a non-clinical sample with 415 trans people aged between 16 and 76 revealed fewer treatment experiences, and fewer contacts with support groups or other trans people among persons from rural areas compared to persons from urban areas (5, 37).

An online survey of 208 health care providers in West Virginia found that although a majority of rural or suburban HCPs held generally positive attitudes about treating trans people, they admitted to assuming their patients to be cisgender and needing further training to effectively offer care to trans people (38). Critically, HCPs who held fewer positive attitudes – who were also shown to be disproportionately male – perceived less barriers to treatment but also showed less personal preference to treat (38). Similarly, a systematic review on health and health care on rural residing SGM have found a lack of favorable attitudes, training and desire to train in rural HCPs (10). According to the U.S. National Transgender Discrimination Survey of 6,436 respondents of all ages, 19% of trans people were ultimately denied treatment by HCPs and 50% had to educate their HCPs about trans-related health care (39). Qualitative interview data from trans women with prior rural residential experience report that in rural areas, trans people have to pay for their own trans-related medications, and hospitals near their homes refuse to care for trans people, even when urgent treatment is needed for self-destructive behavior (29). According to questionnaire data from 13 transgender and sex/gender diverse people (majority aged 25-44), homophobia and transphobia are a factor in health care in the largely rural Northern Territory of Australia (40). HCPs are seen as mostly unhelpful, so several trans people seek medical care in other Australian states (40, 41).

Taken together, this creates a situation in which trans people are faced with (un-)intentional microaggressions and stigma (10, 38) while barriers to care remain largely unaddressed.

### Experiences and Consequences of Insufficient Health Care

As illustrated by case vignette 3 and an answer to the second research question, a negative health care experience can have long-lasting effects and deter trans people from seeking further support, making them more vulnerable to mental and physical problems (42). Results of a systematic review on LGBTQ+ health and health care show that people belonging to SGM and living in rural areas have come to anticipate discriminatory health care based on former experience and often do not trust HCPs enough to disclose their sexual orientation or gender identity (10). As a result, many trans people avoid health care services entirely, which is particularly evident in conservative regions, or are forced to accept what is offered due to a lack of options (10, 36). Often, trans people expect to be treated badly (35) or have already suffered from experiences of discrimination and violence in public institutions, e.g. a doctor’s office or hospital, a mental health clinic or emergency room (39). Analyses of semi-structured interviews with Australian remotely living 15 trans clients aged 19-69 years and 8 HCPs revealed, expected discrimination for several trans people is also based on their conservative assessment of their housing and not necessarily on actual experience (41). Researchers of a U.S. interview study with 10 rural residing trans people (mean age 36.2 years) noted a fundamentally pronounced negative attitude of trans people toward rural HCPs (43).

In a survey of 1,014 U.S. rural residing LGBTQ+ individuals (169 of the respondents were trans with a mean age of 32.2), higher anticipated and experienced stigma correlated with poorer reported health among trans people (44). In particular, assigned female at birth (AFAB) trans people avoid important sexual health services (e.g., contraceptives, PAP tests) and accept health risks if they lack access to specific and trans-informed clinics (45, 46). An illustrative example is offered by HPV vaccination recommendations and HPV vaccination: rural residing US trans individuals are primarily offered treatments based on their sex assigned at birth according to an analysis with 660 LGBTQ+ respondents (ages 18-34; 7% identified as trans man, 4% as trans woman, 6% reported a non-binary gender identity), rather than providing the vaccination to everyone right away (47).

Given the U.S. Survey Behavioral Risk Factor Surveillance System, 237 trans men compared with 163,685 cis adults of all ages showed a reduced likelihood of having a personal family doctor or undergoing cholesterol screening (48). Care refusal is a critical issue, as trans people in rural areas show several health risk factors, such as binge drinking, smoking, substance abuse in general, and higher odds of posttraumatic stress disorder (3, 44, 49).

On average, trans people have significantly poorer mental health than the rest of the general population (39, 50). This is particularly true for residents in rural areas. Research comparing location categories found that trans high school students in rural areas show the highest level of self-injury and suicide attempts (22). However, members of the suburban trans population showed the highest levels of depressive symptoms and suicidal thoughts despite their proximity to larger cities and resources, suggesting that location categories need more differentiation than just “rural” and “urban” (22). As an online survey of 414 trans people (mean age 39.58) found, trans people living in more rural US states suffered more from anxiety and depression than trans peers in other regions (51). In the Trans Health Survey of 902 trans people from Canada and the U.S. (mean age 32.47), rurality correlated with higher social anxiety among trans people (52). In an online survey (mean age 36.0 years), 91 trans people in the U.S. state of Nebraska reported higher rates of discrimination, depressive symptoms, and suicide attempts compared to 676 lesbian, gay, and bisexual people (53). Further, there are strong differences in access to health care between urban and rural citizens according to a survey with 414 trans people aged 18 to 78 years across the U.S. (51). Suitable mental health services are hardly available for rural residing trans people. Even in urban areas, a bottleneck situation exists due to the low number of trans-informed HCPs. The majority of trans people from one region seek treatment from the same few highly specialized experts, most of whom are known by name in the community and play an important gatekeeping role in health care, as highlighted in the European Trans Health Survey and qualitative individual interviews with 10 U.S. MHPs (mean age 56.4) about the health care situation (35, 54).

In a recent qualitative interview study of 2021 with 61 adult transgender and gender diverse people and 23 HCPs of all ages 18 and older, all from 25 different rural U.S. counties, trans people cite urgent community mental health needs such as, in addition to moving away from binary settings, increased accessibility of MHPs through more flexible solutions such as e-health approaches (55). HCPs of the same study did not come up with this idea, focused on existing approaches and systems in ensuring mental health care, but criticize that rural care cannot keep up with urban centers for health care (55). Meanwhile, qualitative interview data from Australia and the United States show that the Internet has become an important resource of information and community building for trans people seeking help (41, 43).

## Discussion

### Evaluation of the Scoping Review

The current scoping review focused on the issues facing trans people in remote, rural, and suburban areas concerning their specific barriers to accessing trans-related health care, and potential approaches to address issues related to place of residence. Several research gaps emerged regarding the impact of location on the health and quality of life of trans people. Through the initial search strategy, it became obvious that previous research has not collected sufficient data on the urban-rural divide in trans health. Many studies did not include rural or suburban trans people at all and could therefore not be considered for the review. Nevertheless, it was possible to obtain an overview of the situation of remotely living trans people by means of a scoping review.

Related to the first research question, we found that living in remote, rural, or suburban areas can significantly impede access to specialized trans health services. Trans-informed HCPs are predominantly found in metropolitan areas, which means long, costly commutes for trans people (2, 21). In addition, many rural or suburban residing trans people experience discrimination from non-specialized or dismissive HCPs in their area (31–33). When we look at the aggravating socioeconomic factors such as financial insecurity, job insecurity, housing insecurity among several trans people, a very limited access to health care becomes apparent (11, 17–19). Thus, according to the first research question, the health burden of trans people in rural areas seems to lie in structural problems related to remoteness, education, housing, health insurance coverage or employment, and age-specific mobility problems that impede access to trans-related health care. However, when evaluating research on urban-rural disparities, it appears relevant not to ignore the substantial number of trans people who have ever experienced homelessness in their lives, and thus may not even be reached by residence-based studies.

We were able to observe this picture also in the answer to the second research question. Starting with even more pronounced bullying experiences at school and in the surrounding area compared to urban trans peers, rural and suburban residing trans people experience discrimination from an early point in their lives (22–24). Discrimination can persist in close-knit rural social structures, including through other forms of discrimination, such as racism (30). Also, cis-heteronormative attitudes and behaviors of HCPs can make goal-directed health care difficult (38, 39). Many rural and suburban residing trans people avoid social contact and do not seek help and support, which can then be reflected in a rejection or avoidance of health care (35, 36). According to the second research question, we conclude that negative experiences and confrontation with strong homophobia and transphobia can damage a trans person’s trust in their own environment. This unfavorable starting point overlaps with already known barriers to accessing trans-related health care, such as long journeys to specialized transgender clinics, and exacerbates the difficult mental health situation of trans people. Trans-related health care is primarily located in metropolitan areas. However, if trans people generally do not expect support from their environment, they may be unaware of support services or avoid trans-related health care services. The isolation of trans people can therefore deepen in rural areas.

In the third research question, we also looked at possible solutions that could address the problems of trans people, especially in rural or suburban areas. We identified that supportive caregivers (27) or SGM community groups (25) are significant in helping a trans person feel safe and comfortable in their environment. Diversity-aware trainings for HCPs and other professionals such as teachers or social workers should also remain in focus to create an inclusive environment (27, 28). In response to the third research question, we identified the often-emerged importance of the “family of choice” for a positive self-image and being accepted in a community (29). Because families of choice often first emerge in adulthood, we as a study team see the need for support systems in general but specifically in rural areas, from early in life. Through LGBTQ+ empowerment groups, education of families of origin, educational and health submissions about diversity, a social climate can be created in which trans people feel accepted and respected. These are meaningful health prevention interventions. These are known aspects that can increase a trans person’s confidence in others and could increase the possibility that they will seek help and support in trans-related health care. New opportunities such as e-health approaches by trans-informed HCPs could fill a gap in care. The literature has barely touched on this possibility, if at all. We would like to illustrate below the potential role of e-health in overcoming or reducing barriers to trans-related health care.

### The Role of E-Health

With the advent of digitalization in private life and the health care system, a number of possibilities are opening up for trans people in terms of individual access to trans-related health care. Qualitative interview data from Australia, North Queensland, revealed that the internet is crucial for many trans people to share resources and compensate for low local networking and peer support. For trans people, the internet and social media play an important role in finding information about gender identity, transition, and trans-related health care, and in reducing isolation, making reliable internet access essential for mitigating or overcoming existing problems, particularly in rural areas (41). Therefore, in light of previous articles and assessments, expanding online (and offline) trans support groups (41) as well e-health approaches (3, 4) could have a beneficial effect on trans-related health care, reduce financial burdens, and optimize the internet’s health care potential. Trans people themselves also expect e-health approaches to improve health care in terms of better accessibility and flexibility (55).

Yet, there is limited data on the use of e-health approaches for LGBTQ+ health care. A pilot study on an e-health program for trans women of color in Washington, DC, appears to be a promising effective and low-cost way to overcome multiple health care barriers and increase the intention to seek trans-related health care (56). Due to the COVID-19 pandemic, many health care services have had to rapidly implement e-health technologies to ensure continued treatment. One U.S. LGBTQ+ clinic has received positive reactions from patients for their video consultations and reports fewer cancellations and no-shows, presumably due to the increased flexibility and decreased effort of attending an appointment. Patients also seemed more comfortable and relaxed (57). Other research on HIV prevention in LGBTQ+ populations *via* e-health has shown promising results in terms of program adherence and satisfaction (58).

E-health in medical care has been used widely in cancer prevention and care, an area comparable to trans-related health care in its specialization and individualization of treatment. A review of e-health programs for cancer care has shown numerous benefits such as increased access to specialist and multidisciplinary health care (59). E-health were as effective as in-person interventions in providing psychosocial support, increasing quality of life and ensuring patient satisfaction (59, 60). Such findings hint toward the potential of e-health for improving health disparities and removing access barriers like the ones discussed in this article. With this in mind, we have designed an e-health program *i^2^TransHealth*, for which we are currently evaluating its efficacy within an RCT (https://clinicaltrials.gov/ct2/show/NCT04290286).

### Research Bias Towards Big Cities and Neglect of Remote Life

The idea that the city is more inclusive than the country is pervasive and most research generally assumes metropolitan areas to be more progressive and trans-inclusive. Some researchers refer to this as the “metronormativity bias” (30). Many trans people and other LGBTQ+ representatives also strive for a life in a big city (12). Trans people themselves expect rural regions to be more conservative and discriminatory, which they often base not on actual experience but on less queer and trans visibility in rural areas (41). However, rural communities are not necessarily negative for trans people, just different (26). Personal fit greatly affects the experience of trans people in rural areas: Some trans men easily find connection in these communities, but this mainly applies to those who fulfill the traditional role of working-class White men, which excludes People of Color and non-traditionally male presenting trans men (30). Perceptions of rural communities can shift depending on life experiences and possibility for comparison with urban areas, as illustrated by a Canadian qualitative study (12). However, the study also showed a strong consensus of almost unequivocal experience of rural socialization as limiting and conservative.

Unfortunately, differentiated group analysis by place of residence often fails because trans people from rural areas are more difficult to reach for studies (22). Due to this limited available data, some reviews only include data on trans people in urban spaces or combine samples from larger cities and suburban regions, which reinforces the already existing metronormativity bias (22, 30). To enable analyses, some studies also combine rural and suburban communities (52). Other researchers strongly advocate differentiating suburban from rural communities, which often results in a very small number of rural participants (12, 18). These varying definitions and operationalizations, or lack thereof, of location categories (e.g., metropolitan regions, non-metropolitan areas, small metropolitan areas, small towns, rural communities, etc.) limit the conclusions that can be drawn from the current research (26). Grouping study participants by postal code or self-classification seems to be common, but a closer look at population sizes could be beneficial for more complex analyses (10).

Thus, reliable comparisons between rural and urban health care conditions remain difficult, although the finding that trans people in rural areas are likely to suffer from greater health inequalities seems fairly robust (9, 10). These inequities are already impacting primary care treatment, disease prevention, and health-damaging behaviors (9), with far-reaching consequences for health care utilization.

### Limitations

A major limitation of the scoping review is the overwhelming representation of North American studies (i.e., 39 studies), largely due to the general lack of research on remotely living trans people. This is also reflected in the fact that previous work from other geographic regions, such as Europe, Africa, Asia, Australia, or South America, has limited in-depth comparison of rural and urban structures for trans people’s lives. The few reports from other regions were added by the study team itself based on its own expertise (i.e., 4 European studies). However, the initial search also yielded at least two Australian studies. Overall, sparse research in trans-related health care has addressed the impact of the variable of place of residence on trans people’s life satisfaction and the quality of health care they receive. This reduces the number of potentially relevant studies and exposes other inequities, such as that trans people who live near larger cities or trans-affirming spaces are more likely to be researched because they are easier to reach for study recruitment. As a study team, we executed the search strategy with the goal of high sensitivity to capture relevant studies that advance transgender- and rural-related research. Therefore, the scoping review depends on the available research literature on the concept of remote, rural, or suburban regions and their intersection with barriers to accessing trans-related health care. The number of relevant studies is small but highlights all the more the urgent need for further empirical research on the marginalization of remotely living trans people.

The focus on rural socialization of currently or ever remotely living trans people varied across studies. With 21 studies, about half of the included studies adopted purely rural or suburban samples, while the other included studies analyzed mixed samples with individuals of different places of residence. In this regard, the mixed samples were unbalanced, considering individuals with rural or suburban residences with their partial low representation in the small percentage range. Generalizing statements across communities are therefore not applicable. An international in-depth comparison of the situation of remotely living trans people is also not applicable due to the divergent study designs and overrepresentation of U.S. studies. On a positive note, as far as we know our scoping review is the first to include Australian, European, and Canadian studies in the literature related to the specific topic of the review.

As a consequence of the concept of a scoping review compared with a systematic review, the results underwent a synthesis of content rather than a critical examination of study design and strength of evidence of the included papers. Because this is one of the first reviews in the context of remote, rural, and suburban areas to highlight potential new solutions such as e-health approaches as potentially improving care for hard-to-reach populations of trans people, a more in-depth look at the global situation of trans people was not possible. The results can be considered preliminary. They should be revised as the implementation of trans-specific support groups and educational opportunities in rural areas, as well as the digitalization of health care, continues to change the influence of the variable of place of residence and allows remotely living trans people to not feel disconnected and to be well served by e-health approaches.

Research on the health of SGM, particularly trans people, specifically in rural areas is very limited and consists mainly of cross-sectional surveys and qualitative studies (10). The definition of “rural” varies and this lack of consistent differentiation between location categories (i.e., rural, suburban, small town, etc.) might disguise distinct aspects that affect health care. Although the particular circumstances in the presented case vignettes of *i^2^TransHealth* may differ, all three trans persons are united by their remote living situation and their difficulties in accessing trans-related health care. Although the access barriers to trans-related health care are well known, it is still difficult for trans-informed HCPs to address structural factors, such as place of residence (11, 19, 35, 39). Clearly, one necessary step is to improve the quality and options of treatment in rural areas by proliferating the expertise currently only available in a few specialized urban clinics.

Thus, competence training for (rural) HCPs and, ideally, also teachers and social workers is of high importance (22, 31, 41) and should cover an intersectional view of discrimination as well as the effects of microaggressions on the course of treatment (34). E-health could also prove a powerful tool for spreading expertise and making quality trans-related health care more widely available. E-health could help break down some of the reservations toward trans-related health care, which stem from the fear of encountering uninformed and even discriminatory HCPs (10, 35, 43). Anecdotal evidence – such as our third case vignette – as well as empirical evidence suggests that these fears are not unfounded: 33% of respondents in the U.S. Transgender Survey said they had had at least one negative experience with a HCP in the past year related to being trans (19).

## Conclusion

Using three case vignettes as a starting point, the scoping review focused on the lives of trans people in remote, rural, or suburban areas. The interest in assessing previous research was how living in remote, rural, or suburban areas affects individual access to trans-related health care services. Having identified place of residence as a potential aggravating factor, we also strove to pinpoint other barriers and risk factors. During the review we discussed intersections of trans mental health and discrimination (11, 39) with age, race, gender expression, geographic location, community size, socioeconomic status, experiences and attitudes towards HCPs. Potential innovative solutions to reduce inequities in access to trans-related health care, in our view, lie in e-health approaches that require further evaluation. Alongside this, HCPs are encouraged to engage the immediate environment, such as family or friends of a trans person, in therapeutic approaches and educational programs on inclusion and diversity to change discriminatory attitudes in communities. This could reduce problems resulting from place of residence and empower individuals to seek help in trans-related health care.

Although research on currently or ever remotely living trans people to access the urban-rural divide in trans-related health care is still in its infancy, a growing field of research has gradually emerged over the past few years. Note that researchers should not merely look at the (un-)availability of resources – e.g., no visible representation of LGBTQ+ groups in rural schools (22) or cis-oriented sexual health services in rural areas (41, 46) – but assess whether they are appropriate and queer/trans-specific (25). Also, a rural setting should not be categorically viewed as a barrier, but as a potentially aggravating factor. For even in a supportive rural environment, long journeys to specialized transgender clinics remain. Therefore, e-health approaches such as our *i²TransHealth* project could be a way to address or mitigate structural barriers. HCPs must widen their scope in order to reach people regardless of their place of residence. This would be one critical step towards decreasing inequalities and reducing the mental, physical, social, and economic burden that trans people have to bear. Whether such approaches are effective in breaking down barriers thus remains a worthy and important topic that we ourselves and hopefully other researchers and HCPs will continue to investigate.

## Ethics Statement

The study was conducted in accordance with the declaration of Helsinki and was approved by the ethics committee of the Hamburg Medical Association (PV7131). All three persons introduced by the case vignettes signed their informed consent. Written informed consent was obtained from the individuals in the case vignettes presented for the publication of any potentially identifiable images or data included in this article.

## Author Contributions

JR: conceptualization, methodology, investigation, writing – original draft, writing – review & editing. WB: investigation, writing – review & editing. LT: investigation, writing – review & editing. AD: writing – review & editing, supervision, project administration, funding acquisition. PB: writing – review & editing, supervision, project administration, funding acquisition. TN: conceptualization, methodology, resources, writing – review & editing, supervision, project administration, funding acquisition. All authors contributed to the article and approved the submitted version.

## Funding

i²TransHealth is funded by the Innovation Committee at the Federal Joint Committee (G-BA) (funding code: 01NVF17051).

## Conflict of Interest

The authors declare that the research was conducted in the absence of any commercial or financial relationships that could be construed as a potential conflict of interest.

## Publisher’s Note

All claims expressed in this article are solely those of the authors and do not necessarily represent those of their affiliated organizations, or those of the publisher, the editors and the reviewers. Any product that may be evaluated in this article, or claim that may be made by its manufacturer, is not guaranteed or endorsed by the publisher.
