# Supplementary material for: Barriers to Accessing Health Care in Rural Regions by Transgender, Non-Binary, and Gender Diverse People: A Case-Based Scoping Review
Source: Front Endocrinol (Lausanne). 2021 Nov 18;12:717821. doi: 10.3389/fendo.2021.717821 (PMC8637736; doi:10.3389/fendo.2021.717821)
Supplement: Supplementary file 1 [file DataSheet_1.docx]

**Appendix 1**

**Identification of studies via databases**

Articles removed *before screening*:

Duplicate records removed (*n* = 133)

Search period: all citations until 2021/08/18 using transgender related AND rural related terms as a search strategy framework

Records identified from:

PubMed (*n* = 187)

Web of Science (*n* = 310)

**Identification**

Articles screened

(*n* = 364)

Articles excluded after title/abstract screen*

(*n* = 287)

Articles assessed for eligibility

(*n* = 77)

44 Articles excluded after full text screen:

Full text not available (*n* = 2);

Irrelevant to research objectives (*n* = 6);

Same databases, overlap (*n* = 2);

Article deals with a general population sample or general LGBTQ+ sample, too trans-nonspecific (*n* = 34)

**Screening**

Articles identified

(*n* = 33)

Additional articles included:

6 articles sourced from reference lists

6 additional sources meeting the criteria based on the expertise of study team

Articles included in synthesis

(*n* = 45)

**Included**

*Figure 1*. Flowchart showing the selection process for the scoping review. * Excluded articles by title/abstract screen (*n* = 287): e.g., due to irrelevant to the focus of the scoping review; the term "rural" was used only once or rarely, or rural socialization was not the focus of the article; trans people were mentioned as part of the LGBTQ+ community, but their concerns were not specifically addressed.
