# Supplementary material for: Barriers to Accessing Health Care in Rural Regions by Transgender, Non-Binary, and Gender Diverse People: A Case-Based Scoping Review
Source: Front Endocrinol (Lausanne). 2021 Nov 18;12:717821. doi: 10.3389/fendo.2021.717821 (PMC8637736; doi:10.3389/fendo.2021.717821)
Supplement: Supplementary file 2 [file DataSheet_2.docx]

**Appendix 2**

Table 1. *Scoping review summary of included studies.*

| Study | Year | Location | Data collection | *n* | Findings |
| --- | --- | --- | --- | --- | --- |
| Aaron & Rostosky | 2019 | USA, Central Appalachia | Qualitative: In-depth interviews about perceptions of maternal support | 25 transgender-identified adults | - Rural focused sample - Trans people attribute a key role to their mothers in influencing the behavior of other family and community members toward themselves |
| Abelson | 2016 | USA, Southeast and Midwest | Qualitative: Intersectional analysis of interviews | 45 transgender men | - Place of residence: 20% lived in rural, 34% in suburban and 47% in urban settings with residential experience in rural areas - Importance of fitting in white rural working-class heterosexual masculinities for rural residing people: For rural life trans men must actively resist pressures of metronormative imperative; must confront themselves with claims to sameness, racism, masculine role expectations, often living as stealth (i.e., being trans is not known in rural public); |
| Bednarczyk et al. | 2017 | USA | Quantitative: Cross-sectional survey on HPV vaccine recommendations and uptake | 660 rural residing LGBT young adults (7% trans man, 4% trans woman, 6% reported non-binary gender identity) | - Rural focused sample - Different HPV vaccine recommendations and uptake more based on sex assigned at birth (cis-female recommendation 46.2%, uptake 43.1%; trans men recommendation 41.9%, uptake 36.6%; cis-male recommendation 17.5%, uptake 14.2%; trans women recommendation 5.3%, uptake 5.3%; AFAB^a^ non-binary recommendation 41.9%, uptake 62.1%; AMAB^b^ non-binary recommendation 33.3%, uptake 20.0%) |
| Brown & Jones | 2014 | USA | Quantitative: Veterans Health Administration (VHA) encounter data from multiple sources | 5,135 transgender veterans | - National sample without percentage information on place of residence - Odds of Black transgender veterans to live in a rural area was 65% less than that of White transgender veterans (AOR=0.35, CI=0.27–0.46, *p*<0.0001) |
| Bukowski et al. | 2017 | USA | Quantitative: Patient socio-demographic information diagnosis data | 5,072 transgender veterans | - Place of residence: urban area (88.1%), large rural city/town (6.2%), small/isolated rural town (5.6%) - Transgender veterans in small/isolated rural towns were more likely to have tobacco use disorder and post-traumatic stress disorder compared to their urban transgender peers |
| Clark et al. ^f^ | 2017 | Canada | Quantitative: Canada Trans Youth Health Survey | 923 trans youth | - Place of residence: 9.3% lived in rural settings, which report very specifically on transportation barriers to accessing care in urban areas - 70.3% had regular family doctor; ↑ general/mental health for persons whose transgender identity/expression was known to the family doctor, different reasons for foregoing care (i.e., barriers to care) |
| Crissman et al. | 2017 | USA | Quantitative: Demographic data from state-based health survey in USA | 691 transgender adults (compared to 150,765 non-transgender) | - Trans people are no more likely or less likely to live in rural areas than cis people (28.7% vs. 22.6%) - Trans people are more likely to be non-White (40.0% vs. 27.3%), below the poverty line (26.0% vs. 15.5%), and are less likely to have a college degree (35.6% vs. 56.6%) |
| Edmiston et al. ^f^ | 2016 | USA | Systematic Review | N/A | - US based review of studies on primary care preventative health services for transgender and gender non-conforming populations - criticizes gaps in literature on transgender people living outside of large coastal urban centers |
| Eisenberg et al. | 2019 | USA, Minnesota | Quantitative: Statewide school-based survey | 2,168 transgender/gender diverse (TGD) high school students | - Place of residence: 16.6% city, 44.8% suburban, 23.2% town, 15.4% rural - ↑ Bullying, ↑ Victimization, ↑ emotional Distress in rural locations |
| Eliason et al. | 2004 | USA | Quantitative: Survey on treatment counselor’s attitudes about LGBT Clients | 351 treatment counselors from urban (Chicago, *n*=109) and rural (Iowa, *n*=242) regions | - Insensitive to LGBT persons in both regions - Little formal training on the needs of LGBT clients - Almost half reported negative or ambivalent attitudes |
| European Union Agency for Fundamental Rights ^g^ | 2020 | Europe | Quantitative: Online EU-LGBTI II Survey | A total of 139,799 LGBTI people, sample size (unweighted): 137,508 people, among them 19,445 trans people | - Place of residence of trans people: 39% big city, 13% suburbs, 34% town/small city, 12% village, 2% farm or home in the countryside - 16% of respondents felt discriminated against by health/social care staff in the 12 months prior to the survey |
| Eyssel et al. ^c,g^ | 2017 | Germany | Quantitative: Online questionnaire about trans health care | 415 trans-identified people | - Place of residence: 26% rural area, 14% city, 30% large city, 30% metropolis - Non-clinical sample - Persons from rural areas showed fewer treatment experiences with transgender health care (*p* < .05), fewer requirements towards transgender health care (*p* < .001), fewer contacts with support groups (*p* < .01) or other trans people (*p* < .001) |
| Fisher et al. | 2014 | USA, Nebraska | Quantitative: Survey on social determinants of health and basic health outcomes | 770 LGBT people, among them 78 transgender people (10.9%) | - Place of residence: In a rural U.S. state 89.5% resided in urban and 10.5% in rural area - ↓ social engagement, ↓ coming-out, ↓ self-acceptance, ↓ health insurance in rural LGBT people - poorer health outcomes for transgender, e.g., higher binge drinking rates |
| Garcia & Crosby | 2020 | USA | Qualitative: Interviews on health services for transgender women in Oregon | 25 transgender women | - Health care for trans people is described as relatively trans-friendly in Oregon - Oregon attracts transgender women from rural, conservative areas - In rural areas, people had to care of transition-related medications on their own, lived in isolation, and had to deal with family violence on their own |
| Grant et al. ^g^ | 2011 | USA | Quantitative: National Transgender Discrimination Survey | 6,450 transgender and gender non-conforming participants | - National sample without percentage information on place of residence - Likelihood of having a household income of less than $10,000/year nearly four times that of the general population - 19% were denied health care, with even higher numbers among people of color - 28% had been harassed in medical facilities and 2% had been victims of violence in medical offices - 50% had to educate their medical providers about transgender care |
| Harb et al. | 2019 | USA, rural Midwest state | Mixed Method: Survey on knowledge and awareness of HPV and Pap tests; Interview about health care system experiences | 17 transgender / genderqueer AFAB^a^ people | - Rural focused sample - Generally positive experiences, but frequent concerns about finding clinics and health care providers who understand the health needs of transgender/genderqueer AFAB^a^ people |
| Heng et al. | 2019 | Australia, North Queensland | Qualitative: Interviews about transgender health | 23 participants (15 trans people, 8 clinicians) | - Sample focuses on a largely regional, rural, or remote Australian state - Importance of support from community, family, friends, and peers for mental health of trans people living in regional areas - Expected discrimination based on anticipations due to regional location rather on actual experiences - Person-centered clinician = one of the most important factors for client’s health care experiences - Internet relevant for finding information, reducing isolation and promoting acceptability and validation of the trans identity |
| Holt et al. | 2020 | USA, Central Great Plains | Qualitative: Interview on mental health care for rural residing transgender and gender non-conforming people | 10 mental health care providers | - 2 reported living in a rural area, 8 reported living in an urban area - finances and insurance as substantial barriers, gate-keeping role of mental health care providers |
| Horvath et al. | 2014 | USA, Minnesota | Quantitative: Survey on mental health, substance use and sexual risk behaviors | 1,229 self-identified rural and non-rural transgender adults | - Rural residing: 214 trans women, 130 trans men; especially great barriers to care for trans men residing in rural areas - ↓ mental health in rural trans men (highest scores in Brief Symptom Inventory, BSI); ↑ marijuana use; ↑ unprotected sex among trans women |
| Hughto et al. ^f^ | 2016 | USA | Quantitative: Data from cross-sectional survey to analyze geographic and individual factors associated with health care access | 5,831 transgender adults | - National sample without percentage information on place of residence - age, gender, race, income, and health care avoidance associated with care refusal - percent of state residents voting Republican = strongest state-level predictor of care refusal |
| Hulko & Hovanes | 2018 | Canada, BC | Qualitative: Interviews and focus groups on experiences of sexual and gender minorities in a small city or rural town | 13 LGBTQ youth, among them 5 transgender people (3 FtM^d^, 2 MtF^e^) | - Rural focused sample - Discussed class, age, white privilege - limitations of small cities are more reported by people who lived only in rural areas - difficulties for trans people to explain their identity |
| James et al. ^g^ | 2016 | USA | Quantitative: U.S. Transgender Survey (USTS) | 27,715 respondents | - National sample without percentage information on place of residence - ﻿30% of respondents have experienced homelessness at some point in their lives, ﻿12% experienced homelessness in the past year - 25% of respondents had a problem with their insurance in the past year - 55% of those who sought coverage for transition-related surgery in the past year were denied, and 25% of those who sought for coverage for hormones in the past year were denied - 33% of those who saw a health care provider in the past year reported at least one negative experience - 23% of respondents did not see a doctor when they needed to because they were afraid of being mistreated as a transgender person - 33% did not see a doctor when they needed to because they could not afford it |
| Kano et al. | 2016 | USA, New Mexico | Qualitative: Data of town hall dialogues and a summit for stakeholders’ recommendations | 207 LGBTQ people | - Rural focused sample - Isolation of youth, elders and transgender people in largely rural, multicultural state of New Mexico - need for trainings for health care providers in remote regions due to their low LGBTQ patient bases and knowledge |
| Kaplan et al. | 2019 | Canada/USA | Quantitative: Trans Health Survey | 902 transgender and gender non-conforming (TGNC) people | - Place of residence: urban (*n*=443), suburban (*n*=264), small town/rural (*n*=195) - ↑ social anxiety in small-town/rural environments; social support, TGNC community connectedness, and TGNC pride associated with lower social anxiety |
| Kauth et al. | 2017 | USA | Quantitative: Health Survey | 252 lesbian, gay and transgender veterans, among them 84 transgender women (33.3%), 24 transgender men (9.5%) | - Place of residence: rural/small town (*n*=70), suburban/urban (*n*=182) - for trans people little to no health differences between suburban/urban vs. rural/small town (e.g., depression, anxiety scores regardless of place of residence), however longer trips to primary care providers |
| Kerry | 2017 | Australia, Northern Territory | Quantitative: Survey on health needs of trans people | 13 transgender and sex/gender diverse people | - Rural focused sample - Seeking medical care in other states because access to trans-informed health care is difficult in largely rural Northern Territory - health care providers were considered unhelpful by a majority of participants - sparsely populated Northern Territory generally creates isolation, limits availability of medical and support services - strong homophobia and transphobia in the region |
| Knutson et al. | 2018 | USA | Qualitative: Telephone/video interviews on recommendations to other transgender people in rural areas regarding health care | 10 transgender people living in rural areas | - Rural focused sample - General bias against rural providers, encouragement to check the competence of health care providers |
| Kosciw et al. | 2009 | USA | Quantitative: National survey focusing on school climate | 5420 LGBT secondary school students, among them 245 transgender people (4.5%), 217 people with other gender identity (4.0%) | - National sample without percentage information on place of residence - female youth were less likely and transgender youth were more likely to report being victimized because of their gender expression than male youth, and older youth were less likely to be victimized for this reason than younger youth - for LGBT youth schools in rural communities were the most unsafe |
| Logie et al. | 2019 | Canada, Northwest Territories | Qualitative: Interviews on sexual health care experiences | 51 people, among them 16 LGBTQ+ youth, 21 LGBTQ+ adults and 14 key informants (e.g., health care providers) | - Rural focused sample - uncertainty on the part of healthcare providers about using non-discriminatory language with trans clients, great need for gender-inclusive care on the part of trans people in rural Arctic Canada |
| Loo et al. | 2021 | USA | Qualitative: Focus groups discussions or in-depth interviews on gender-affirming health care | 61 adult transgender and gender diverse people, 23 health care professionals | - Rural focused sample: 25 rural counties in Massachusetts, New York, Connecticut, Vermont, and New Hampshire - Community members wanted better availability of mental health professionals (flexibility, e.g., via e-health approaches) - HCPs referred more to existing approaches and systems and emphasized the lack of access to mental health services in rural areas compared to urban health care systems |
| O’Bryan et al. | 2020 | USA | Quantitative: Survey and assessment of health-related quality of life | 141 transgender and gender expansive (TG/GE) youth | - Rural focused sample - ↓ mental health measures than general US population, assessed in a rural gender wellness clinic |
| Paceley et al. | 2017 | USA, Midwestern | Mixed Method: Survey and interviews on youth’s demographics and community contexts | 70 transgender youth | - Place of residence: 57% lived in small and nonmetropolitan counties - supportive communities were associated with more available sexual and gender minority resources |
| Paceley et al. | 2020 | USA, Kansas | Qualitative: Interviews on resources, visibility, policies, and ideologies | 19 transgender young people | - Rural focused sample - youths’ community‐ and school‐based experiences impacted by e.g., presence of resources, perceived community climate, past experiences within these spaces, awareness |
| Patterson et al. | 2019 | USA, Tennessee | Quantitative: Survey on LGBT cultural competence and post-survey interviews | 85 nurse and physicians | - Rural focused sample - LGBT microaggressions and microinvalidations present in the clinical setting, e.g., regarding trans people conscious refusal of preferred pronouns or names |
| Rosenkrantz et al. ^f^ | 2017 | USA | Systematic Review | N/A | - US based review on health and health care of rural residing LGBT people - isolation in rural areas and low levels of social support negatively impact LGBT health |
| Rowan et al. | 2019 | USA, West Virginia | Quantitative: Survey on knowledge and attitudes toward transgender patients | 208 health care providers of West Virginia University Hospitals | - Rural focused sample - more than 40% of respondents wished to be better educated about transgender health issues - male health care providers: ↑ negative perceptions of the transgender community; perceived fewer barriers due to personnel |
| Seelman et al. | 2019 | USA | Quantitative: Behavioral Risk Factor Surveillance System Data | 237 transgender men (compared to 163,685 cisgender people) | - Place of residence: city or suburb (83.4% transgender men, 82.6% cisgender), rural area (16.6% transgender men, 17.4% rural area) - transgender men living in rural areas nearly 9 times less likely than transgender men in urban areas to say they have someone they consider to be a personal doctor |
| Sinnard et al. ^f^ | 2016 | USA | Quantitative: Survey to investigate geographic location, anxiety, and depression | 414 transgender people | - State-specific evaluation - ↑ anxiety and ↑ depression in rural/conservative geographical locations - Disparities in access to healthcare exist between urban and rural areas as well as broader geographic regions of the US |
| Smiley et al. ^g^ | 2017 | Europe | Quantitative: Trans Health Survey in Georgia, Poland, Serbia, Spain, and Sweden | 885 trans health care users, 888 health care providers | - European sample without percentage information on place of residence - Common reasons for not seeking help: fear of prejudice on the part of health care providers (44.1%) and lack of trust in the services offered (41.1%) - 55.8% delay seeing a doctor for general health care because of their gender identity - Partially stricter perceptions of health care providers regarding diagnosis and pathologization, informed consent, and legal gender recognition |
| Smith et al. | 2018 | USA, Montana | Qualitative: Interviews on well-being, mental health, suicidality | 30 transgender adults | - Rural focused sample - Bullying, discrimination, marginalization especially in rural areas as additional risks, high suicidality in rural areas |
| Stewart et al. | 2020 | USA | Quantitative: Retrospective chart review of participants | 255 transgender and gender diverse patients | - Rural focused sample - ↓ contraception use and ↓ cervical cancer screening in a rural academic center - trend toward lower utilization rates in rural cohort compared to urban settings |
| Su et al. ^f^ | 2016 | USA, Nebraska | Quantitative: Survey on mental health disparities | 767 LGBT, among them 91 transgender people | - Place of residence: In a rural U.S. state 90.4% transgender/89.1% non-transgender resided in urban and 9.6% transgender/10.9% non-transgender in rural area - ↑ discrimination, ↑ depression, attempted suicides in transgender people of rural Nebraska compared to LGB people |
| Walinsky & Whitcomb | 2010 | USA | Qualitative: Focus group and individual interview | 7 rural residing trans people | - Rural focused sample - Counselors should be specifically knowledgeable about issues of vocation, personal change and coming out, acceptance, and identity in order to be better advocates for trans people |
| Whitehead et al. | 2016 | USA | Quantitative: Survey on outness, stigma and primary health care utilization | 1014 rural residing LGBT people, among them 169 transgender and non-binary people | - Rural focused sample - ↑ stigma in transgender and non-binary participants - anticipated and enacted stigma scores associated with lower self-reported health score - transgender and non-binary people more likely to travel longer distances to receive care |

^a^ AFAB = assigned female sex at birth

^b^ AMAB = assigned male sex at birth

^c^ see also Koehler et al. (2018) ^g^ with an analysis of the urban-rural comparison

^d^ FtM = female to male

^e^ MtF = male to female

^f^ additional articles sourced from reference lists (see Figure 1, Supplementary Material)

^g^ additional sources meeting the criteria based on the expertise of study team (see Figure 1, Supplementary Material)
