# Supplementary material for: Barriers to Accessing Health Care in Rural Regions by Transgender, Non-Binary, and Gender Diverse People: A Case-Based Scoping Review
Source: Front Endocrinol (Lausanne). 2021 Nov 18;12:717821. doi: 10.3389/fendo.2021.717821 (PMC8637736; doi:10.3389/fendo.2021.717821)
Supplement: Supplementary file 3 [file DataSheet_3.docx]

**Appendix 3: Keywords and search strategies used for the scoping review.**

**Concepts/Keywords**

**Transgender related terms**

transgender, transsexualism, gender dysphoria, gender incongruence, gender identity disorder, non-binary, gender diverse, genderqueer, gender fluidity, gender non-conforming, agender, genderless

**Rural related terms in relation to place of residence**

remote, rural, suburban, small city, small town, village, urban-rural divide

**Web of Science**

#1 (((((((((((((ALL=(transgender)) OR ALL=(transsex*)) OR ALL=("gender dysphori*")) OR ALL=("gender incongruen*")) OR ALL=("gender identity disorder")) OR ALL=("nonbinary")) OR ALL=("non-binary")) OR ALL=(“gender diverse”)) OR ALL=(genderqueer)) OR ALL=(“gender fluid*”)) OR ALL=(“gender non-conform*”)) OR ALL=(“gender nonconform*”)) OR ALL=("agender")) OR ALL=(genderless)

#2 ((((((ALL=(remote)) OR ALL=(rural)) OR ALL=(suburban)) OR ALL=("small city")) OR ALL=("small town")) OR ALL=(village)) OR ALL=("urban-rural divide")

#1 AND #2

**Timespan until 2021/08/18: 310 Results**

**PubMed**

#1 (((((((((((((transgender) OR (transsex*)) OR ("gender dysphori*")) OR ("gender incongruen*")) OR ("gender identity disorder")) OR ("nonbinary")) OR ("non-binary")) OR ("gender diverse")) OR (genderqueer)) OR ("gender fluid*")) OR ("gender non-conform*")) OR ("gender nonconform*")) OR ("agender")) OR (genderless)

#2 ((((((remote) OR (rural)) OR (suburban)) OR ("small city")) OR ("small town")) OR (village)) OR ("urban-rural divide")

#1 AND #2

**Timespan until 2021/08/18: 187 Results**
